# The reporting and handling of missing data in longitudinal studies of older adults is suboptimal: a methodological survey of geriatric journals

**DOI:** 10.1186/s12874-022-01605-w

**Published:** 2022-04-26

**Authors:** Chinenye Okpara, Chidozie Edokwe, George Ioannidis, Alexandra Papaioannou, Jonathan D. Adachi, Lehana Thabane

**Affiliations:** 1 Department of Health Research Methods, Evidence and Impact, McMaster University, Hamilton, ON L8S 4L8 Canada; 2 Roche Products Ltd, Ikeja, Lagos, Nigeria; 3 GERAS Centre, Hamilton Health Sciences, Hamilton, ON Canada; 4 Department of Medicine, McMaster University, Hamilton, ON Canada; 5 Biostatistics Unit, Research Institute of St Joseph’s Healthcare, Hamilton, ON Canada; 6 Faculty of Health Sciences, University of Johannesburg, Johannesburg, South Africa

**Keywords:** Missing data, Longitudinal studies, Review, Methods, Older adults

## Abstract

**Background:**

Missing data are common in longitudinal studies, and more so, in studies of older adults, who are susceptible to health and functional decline that limit completion of assessments. We assessed the extent, current reporting, and handling of missing data in longitudinal studies of older adults.

**Methods:**

Medline and Embase databases were searched from 2015 to 2019 for publications on longitudinal observational studies conducted among persons ≥55 years old. The search was restricted to 10 general geriatric journals published in English. Reporting and handling of missing data were assessed using questions developed from the recommended standards. Data were summarised descriptively as frequencies and proportions.

**Results:**

A total of 165 studies were included in the review from 7032 identified records. In approximately half of the studies 97 (62.5%), there was either no comment on missing data or unclear descriptions. The percentage of missing data varied from 0.1 to 55%, with a 14% average among the studies that reported having missing data. Complete case analysis was the most common method for handling missing data with nearly 75% of the studies (*n* = 52) excluding individual observations due to missing data, at the initial phase of study inclusion or at the analysis stage. Of the 10 studies where multiple imputation was used, only 1 (10.0%) study followed the guideline for reporting the procedure fully using online supplementary documents.

**Conclusion:**

The current reporting and handling of missing data in longitudinal observational studies of older adults are inadequate. Journal endorsement and implementation of guidelines may potentially improve the quality of missing data reporting. Further, authors should be encouraged to use online supplementary files to provide additional details on how missing data were addressed, to allow for more transparency and comprehensive appraisal of studies.

**Supplementary Information:**

The online version contains supplementary material available at 10.1186/s12874-022-01605-w.

## Background

Longitudinal studies inherently suffer from missing data due to the multiple waves of data collection that increase the chance of non-response and participant attrition [1, 2]. In studies of older adults, there is high risk for missing data due to the susceptibility of this population to physical and cognitive decline, illness, and death [3], which may impact on completion of assessments. The likelihood of having incomplete observations increases with increasing age. For example, a review of attrition in longitudinal studies in the elderly found a 25% increased risk in drop out rates for every decade increase in age [4]. The presence of missing data in these studies could lead to biased and inefficient estimates that can threaten the validity of study inferences, especially if not properly addressed [5, 6].

The appropriateness of methods for handling missing data largely depends on the extent and mechanism of missingness [7]. The amount of missing data may be considered negligible if less than 5% [8]; however if participants with missing data differ from those with complete data, or where data for key variables are unavailable, the resulting estimates could be biased [7]. Proper handling of missing data requires exploration of the mechanisms of missingness, whereby data can be assumed to be Missing Completely at Random “MCAR”, (where missingness is unrelated to the observed or unobserved data), Missing at Random “MAR” (where missingness can be explained by observed data only) or Missing not at Random “MNAR” (where missingness is dependent on unobserved data) [5, 7]. The assumptions of the mechanism of missingness made for any data entail different approaches for dealing with the missing data. However, there are no techniques that can correctly determine mechanism of missingness [5], and in practice there could be a mix of different mechanisms at play in the data [9]. As such, sensitivity analysis is recommended to test the stability of the results to different assumptions, particularly where there is a strong indication that the missing data is non-ignorable, that is, MNAR [5, 10].

For adequate handling of missing data, existing guidelines [6, 11] recommend comprehensive descriptions of the amount of missing data, reasons for missingness, methods used to deal with missing data and assumptions that were made about the missingness mechanism. Clear and detailed reporting of missing data improves transparency and allows readers to assess the validity and applicability of the study results. However, reviews of clinical and epidemiological studies have shown persistent practice of poor reporting and inappropriate handling of missing data [2, 12–17]. These reviews mostly focused on randomized controlled trials and different clinical areas. Only one review [18] has specifically investigated this issue in aging studies; however, it was limited to publications from six cohort studies. In this paper, we reviewed the extent, current reporting, and handling of missing data in longitudinal observational studies of older adults.

## Methods

### Data sources and search strategy

Medline and Embase databases were searched for studies published from January 01, 2015, to December 31, 2019, to assess the current practice on reporting and handling of missing data. The search strategy was developed with the help of an experienced librarian and included the following key search terms: Longitudinal studies AND Older adults. Initially, no limits were set to identify studies; however, due to the impracticability of reviewing tonnes of records identified, the search was restricted to 10 high-ranking general geriatric journals with the highest impact factor that publish clinical studies [19]. They include Age and Aging, Aging and Disease, Geroscience, Journal of Gerontology: Medical Sciences, Journal of American Geriatric Society, Journal of American Medical Directors Association, BMC Geriatrics, Aging Clinical and Experimental Research, Journal of Aging and Health, and Clinical Interventions in Aging. We also restricted the search to only articles published in English, as it is the only language shared by the reviewers. The search strategy can be found in the supplementary files (Additional file 1).

### Study selection

Abstracts of the identified citations were screened for eligibility based on the following criteria (i) observational, defined as studies that did not include any intervention, (ii) longitudinal, if they had at least one wave of data collection after baseline assessment, and (iii) among older adult population, defined as persons aged 55 years or older. We excluded meta-analyses, randomized controlled trials, study protocols and simulation studies. Conference abstracts were also removed as they were considered too short to have sufficient information on missing data handling. Full texts were randomly selected for review from the pool of eligible studies until the target sample size of at least 139 articles was reached. The sample size was calculated using the formula for a single proportion at 5% precision and 95% confidence level [20], assuming that 90% of studies report missing data based on the average amount from previous reviews [2, 17, 21]. We randomly selected 150 studies per time for review, replacing excluded articles by another round of random selection. This was done twice yielding 165 studies that met the eligibility criteria. Random sampling of studies was performed using an online random number generator (available at: www.random.org).

All abstracts were screened for eligibility by one reviewer while two independent reviewers conducted the full text review and data abstraction. A pilot full text review and data abstraction were performed on 10% of the articles to assess the consistency of reporting between the reviewers. Modifications were made to the aspects that were unclear in the inclusion criteria and data abstraction form. Discrepancies in data collected were resolved by discussion and consensus.

### Data extraction and analysis

We extracted information on study identity (title, author, year of publication and journal name), study setting, study design (prospective or retrospective), duration of follow up, number of data collection waves, method of data collection, sample size, primary statistical analysis method and missing data information. The missing data component was based on the recommended guidelines by STROBE and Sterne et al. (see Table 1 for details). These included: amount of missing data, reasons for missing data, mechanism of missingness, method used to handle missing data and sensitivity analysis if performed. Where multiple imputation was used, we extracted information on whether the following were reported: variables used, number of imputations, evaluation of imputation procedure and handling of non-normal or categorical variables. The highest value of missing covariate or outcome data reported among all variables with incomplete observations were selected instead of adding them together to avoid double counting. Where the amount of missing primary outcome data was not explicitly stated, we determined that by calculating the difference between the number of enrolled participants and number included in the analysis. Online supplementary files were accessed for additional information on dealing with missing data, where it was referenced in the main article.

**Table 1:** General reporting guidelines for missing data

**STROBE Guideline**	
i State the amount of missing data for per variable and analysis step	
ii Provide reasons for missing data	
iii Indicate the number of individuals excluded due to missing data	
iv Describe method used to handle missing data	
v State the assumptions made for missing data analysis	
vi Perform sensitivity analysis to examine robustness of findings	
**Sterne et al (for multiple imputation)**	
i Compare differences between individuals with and without missing data	
ii Indicate number of imputed datasets	
iii State the variables included in the imputation model	
iv Describe how non-normally distributed and categorical variables were handled	
v Evaluate multiple imputation analysis	

Data were summarised descriptively as frequencies and proportions per the reporting and handling of missing data. The review data were managed with Covidence systematic review software (Veritas Health Innovation, Melbourne, Australia, www.covidence.org), and analyses were performed using Stata 13 (Stata Corps, College Station, Texas, USA).

## Results

### Characteristics of included studies

**Fig. 1:**
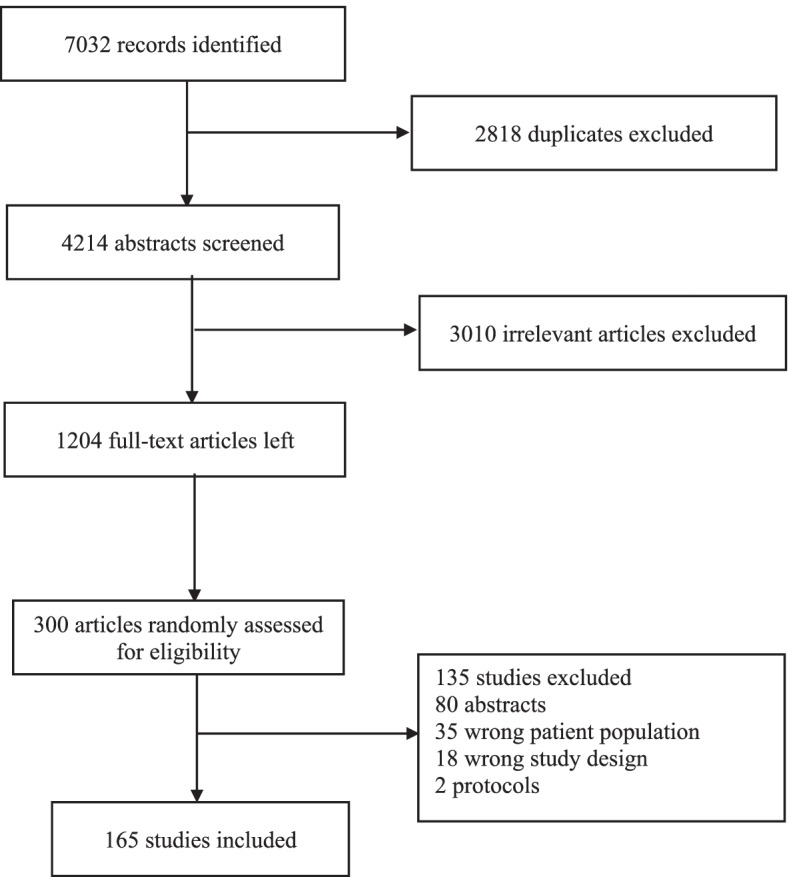
Flowchart of study inclusion process

Figure 1 shows the flowchart of study inclusion process. The search yielded 7032 articles and after 2818 duplicates were removed, 4214 remaining abstracts were screened for eligibility. Of these, 3010 did not meet the inclusion criteria and were excluded, leaving 1204 full-text articles. A random sample of 300 full-text studies were selected for assessment, of which 135 were further excluded. A total of 165 studies were eventually included in the review. The characteristics of the included studies are summarised in Table 2. Overall, majority of the studies were retrospective cohort 119 (72.6%) and conducted at multiple sites 130 (85.0%). Data were collected mostly via surveys 102 (63.9%), over a median of 3 waves and for a median of 44 months of follow-up. The median (IQR) sample size of included studies was 1234 (350–890,544).

**Table 2:** Characteristics of included studies

Description	Total (***n*** = 165)
Study design, n (%)*	
Prospective	45 (27.4)
Retrospective	119 (72.6)
Sample size, Median (IQR)	1234 (350–890,544)
Sample size, n (%)*	
< 1000	69 (43.4)
1000–10,000	65 (40.9)
> 10,000	25 (15.7)
Study site, n (%)*	
Multisite	130 (85.0)
Single site	23 (15.0)
Number of data collection waves, Median (IQR)	3 (3–5)
Duration of follow-up (months), Median (IQR)	44 (12–108)
Method of data collection, n (%)*	
Administrative data	28 (17.4)
Surveys^a^	102 (63.4.9)
Mixed	31 (19.2)

n, number; %, percent; * frequencies do not add up to 165 because some studies did not report these characteristics

^a^includes clinical report form or any study questionnaire

### Reporting of missing data

Details of the reporting of missing data are presented in Table 3. In 79 (47.9%) of the studies, there was either no mention of missing data or unclear statements about it. Among 82 (49.7%) with missing data, the proportion ranged from 0.1 to 55%, with a 14.5% average. About a quarter% (*n* = 21) of these studies stated the reasons for missing data, which were mainly due to lost to follow-up 12 (57.1%). Of the studies that reported having missing data, the majority, 64 (78.0%), described the method used to handle missing data. Only 8 (11.3%) studies specified the type of mechanism of missingness assumed in the analysis. Sensitivity analysis on the methods used for handling missing data was reported in 7 (8.5%) of the studies and the results of this analysis were presented in 4 (57.1) of them. Online supplementary files were used to report additional details on missing data in approximately 3.7% (*n* = 6) of all included studies.

**Table 3:** Reporting of missing data

Description	n (%)
Reported the amount of missing data (*N* = 165)	
Yes	86 (52.1)
No	57 (34.6)
Unclear	22 (13.3)
Reported reasons for missing data (*N* = 82)^a^	
Yes	21 (25.6)
No	52 (63.4)
Unclear	9 (11.0)
Reported number of individuals excluded due to missing data (*N* = 66)^b^	
Yes	55 (83.3)
No	4 (6.1)
Unclear	7 (10.6)
Described method used to handle missing data (*N* = 82)^a^	
Yes	64 (78.0)
No	9 (11.0)
Unclear	9 (11.0)
Stated the assumptions for missing data methods (*N* = 71)^c^	
Yes	8 (11.3)
No	61 (85.9)
Unclear	2 (2.8)

n/N, Number; %, percent

^a^number of studies that reported having missing data

^b^number of studies that excluded individuals based on missing data

^c^number of studies that reported methods for handling missing data

### Handling of missing data

Table 4 shows the methods used to deal with missing data in the studies reviewed. Among studies that reported methods for dealing with missing data (*n* = 70), complete case analysis was the most common method with approximately 75% of the studies (*n* = 52) excluding individual observations due to missing data, at the initial phase as part of the inclusion criteria or at the analysis stage. Seventeen studies (26.2%) where participants were excluded based on data completeness compared those with and without missing data. Other methods used for handling missing data in order of popularity include multiple imputation 10 (14.3%), full information maximum likelihood 3 (4.3%), inverse probability weighting 2(2.8%), single imputation 2 (2.8%) and pattern mixture model 1 (1.4%). For 12 (14.6%) of the studies that reported having missing data, there was no explicit description of the analytical approach used. In eight studies that indicated the mechanism of missingness, the assumptions were MAR in six and MNAR in two. Of the 10 studies where multiple imputation was used, only 1 (10.0%) study followed the Sterne guideline fully, clearly specifying the variable used in the imputation model, indicating the number of imputations, evaluating the model, and reporting how non-normal and categorical variables were handled in the imputation process. Sensitivity analysis was performed in 7 (10.0%) of the studies that reported a method for dealing with missing data. Survival analysis was the most frequently used primary analysis method in 51 (31.5%) of all included studies.

**Table 4:** Handling of missing data

Description	n (%)
Methods used for dealing with missing data (*N* = 70)^a^	
Complete case analysis	52 (74.3)
Multiple imputation	10 (14.3)
Full information maximum likelihood	3 (4.3)
Inverse probability weighting	2 (2.8)
Single imputation	2 (2.8)
Pattern mixture model	1 (1.4)
Compared differences between individuals with and without incomplete data (*N* = 65)^b^	
Yes	17 (26.2)
No	48 (73.8)
Performed sensitivity analysis to test robustness of results (*N* = 70)^a^	
Yes	7 (10.0)
No	60 (85.7)
Unclear	3 (4.3)
***For multiple imputation*** *(N = 10)*^*c*^	
Indicated number of imputed datasets	
Yes	5 (50.0)
No	5 (50.0)
Unclear	0 (0.0)
Reported variables included in imputation model	
Yes	4 (40.0)
No	5 (50.0)
Unclear	1 (1.0)
Described handling of non-normal and categorical variables	
Yes	2 (20.0)
No	8 (80.0)
Unclear	0 (0.0)
Evaluated multiple imputation analysis	
Yes	1 (100)
No	8 (80.0)
Unclear	1 (10.0)

n/N, Number; %, percent

^a^number of studies that reported methods for dealing with missing data

^b^number of studies that excluded individuals based on missing data

^c^number of studies that used multiple imputation

## Discussion

This review shows that the reporting and handling of missing data in longitudinal studies of older adults are suboptimal. Insufficient and unclear reporting, exclusion of participants with missing data, failing to assess the robustness of the missing data results are still common practices. Generally, there is poor adherence to recommended guidelines for reporting and handling of missing data. This is consistent with other reviews of missing data across different research designs and clinical areas [12, 21–24]. Considering that all the articles included in this review were published at least more than 5 years following the release of these guidelines, it was expected that the reporting standards would have improved over time. Guideline endorsement by journals could enhance compliance with standards [25], but only four of the ten included journals mentions one of the guidelines in its instructions to authors.

In some of the studies included, there was no indication of whether data were missing or fully observed. Similar to previous reviews [13, 23], it was unclear how the analytical cohort were selected and how much missing data there was, particularly in retrospective cohort studies. In the absence of comments on missing data, the reader may likely assume that the data were complete, which may either be true or false. Leaving room for speculation falls short of transparent reporting and impairs critical appraisal and replicability of the study. With the 14% average proportion of missing data observed in the studies where it was reported, there is indication that longitudinal studies among older adults are susceptible to a high amount of missing data that are non-negligible.

Where there are missing data, the common practice for dealing with them was complete case analysis, in which individuals with incomplete observations are removed. Methodological reviews of missing data since 2004 have consistently reported similar findings [14, 16–18, 21, 23, 26]. The persistent use of this method may reflect its ease and simplicity, as well as the fact that it is the default approach in most traditional statistical software [5, 23]. Since there are no in-built mechanisms to flag missing data in these applications, they may go unnoticed. Therefore, performing an exploratory analysis to understand the extent of missing data is an important part of the first step in data analysis to address missing data problem.

When complete case analysis is used, the underlying assumption is that missing data are MCAR, implying that the missingness is unrelated to the observed or unobserved data [5, 7]. Simply put, the fully observed sample is still representative of the study population [5]. This assumption is plausible when the amount of missing data is minimal [13]. In the presence of large proportion of missing data, the resulting estimates will not only be inefficient but could be biased [7, 15]. In some of the studies, exclusion of participants with missing data occurred at the initial phase of inclusion in the study. That is, the fully observed dataset reported in these studies were due to some eligibility criteria that defined the sample based on data completeness; potentially to avoid missing data problem. The majority were retrospective cohort studies where a subset of the original population was used. Excluding participants due to missing data at any phase will have same potential for bias if the groups with or without complete data differ systematically [15].

In the context of longitudinal studies of older adults, the use of complete case analysis to deal with missing data may yield biased estimates. With extended duration of observation and multiple waves of data collection, it is unlikely that missing data will be MCAR. Elderly participants are at increased risk of events such as poor or compromised health, hospitalization, institutionalization, and death, that limit their ability to return for a follow up assessment, or complete surveys over time [3, 27]. Consequently, selective attrition may occur, where healthier older adults are more likely to remain at the end of the study [4]. For example, frail older adults are vulnerable to adverse events [28]; as a result, they are less likely to be available to complete study assessments, including frailty measures. In this case, having missing data for frailty or other measurements may be a function of how frail a participant is. Therefore, MAR or MNAR are plausible assumptions to make.

While it may not be feasible to categorically prove the mechanisms of missingness at play in a dataset [5], there are few assessments that could guide our assumptions. A comparison of the baseline characteristics of those with and without complete data could indicate whether missingness is dependent on the observed variables, if the two groups differ significantly [13]. Other methods include Little’s MCAR test [29] or logistic regression to determine the variables that are associated with missing data indicators [30]. However, with MNAR, it will be impracticable to perform any evaluations for unobserved data. Assumptions typically rely on a priori biological, clinical, or epidemiological knowledge and insights on reasons for missing data [15]. Assessments of assumptions were infrequent in this review as in other reviews of observational studies [13, 23]. Regardless of the mechanism of missingness assumed or methods used, it is important to examine the robustness of the results to different alternative assumptions and methods [5, 11]. We found that such sensitivity analysis was performed in only a limited number of studies.

In some of the reviewed studies, the principal analysis involved methods such as survival analysis that handle incomplete outcome data differently. In majority of these studies, there was no mention of missing data and how they were addressed. When participants have unobserved outcome data in survival analysis, they are typically addressed by censoring, where available data are used until the last time of observation [31]. This method may bias the results when the censoring is informative, that is, censored participants have higher or lower risk of experiencing the outcome [3]. Additionally, it could be problematic when dealing with missing covariate values in the presence of time-dependent variables and time-varying effects or when assessing proportional hazards assumptions [23]. Carrol et al. [23] provide detailed descriptions for dealing with missing covariate data when using survival analysis in observational studies.

Multiple imputation was used in very few studies to deal with missing data despite its popularity and its availability in mainstream statistical packages [5, 18]. This method is based on the MAR assumption which is considered valid in many longitudinal data contexts [32]. It involves reproducing multiple complete versions of the original dataset by replacing the missing observations with plausible values, then combining them into a single result [7, 33]. Multiple imputation reflects the uncertainty around missing data prediction compared to single imputation that does not account for the variability around the imputed estimates [6]. Unlike complete case analysis, this method allows for the use of all available data, thus minimizing the loss of precision and power [6, 13].

Where multiple imputation is used, existing guidelines recommend describing elements of the procedure to facilitate review [6], but these details were scantly presented in the studies reviewed. The only study [34] in which the method was described in full used an online supplementary file for that purpose. This allowed for a comprehensive appraisal of the missing data handling in that study. Online supplementary files provide great space for reporting additional study details that could not be presented in the main article due to word or page limits. However, its use for presenting missing data information is uncommon, with only 3% of the studies referring to it in the primary text.

In situations where data are MNAR, that is, the probability of missingness is dependent on the unobserved data [7]; modelling the missing data becomes more challenging and requires more sophisticated techniques. For example, one study that examined the association between cognitive decline and life-space mobility in community-dwelling older adults used pattern-mixture model to account for the probable non-ignorable missingness [35]. In the study, participants who dropped out had lower scores on the predictors, intermediate and predicted variables compared to those who remained, which is suggestive of non-random missingness. Pattern-mixture model allowed for modelling participants’ missingness and response within each missing data pattern [36]. Selection model could also be used to handle non-random missing data by modelling the probability of participants’ responses and missing values based on a common selection factor [32].

### Limitations

Our survey is limited by several restrictions applied in the search. It is possible that some studies may have been missed due to the limitations of the search to few general geriatric journals. In addition, we randomly selected studies for data abstraction as it was impractical to include all eligible articles. Notwithstanding, we expect that the practices described in this review provide a snapshot of the actual practice in the entire field. Further, we did not exclude studies from the same cohort of participants, so there may be duplication or overlap of data or reporting, particularly for retrospective cohort studies. Since this review was restricted to observational studies of older adults, the current practice in handling and reporting of missing data for other research designs, such as randomized controlled trials may differ.

## Conclusion

Inadequate reporting and lack of rigour in handling of missing data are prevalent in longitudinal observational studies of older adults. The susceptibility of this population to missing data makes it imperative for the issue to be addressed adequately where present. However, authors either do not mention it or at best exclude participants with missing data. These have implications on study validity and transparent reporting. Progress in the implementation and compliance with reporting standards could be enhanced with endorsement of the recommended guidelines by journals. In addition, authors can take advantage of the underutilised online supplementary files to provide details of missing data analysis. It is worth noting that better reporting on missing data handling is associated with higher citation counts [14], which could potentially improve the utilization and contribution of these studies. Please see supplementary file 3 for guide on reporting of missing data and examples of standard reporting.

## Supplementary Information

**Additional file 1.**

**Additional file 2.**

**Additional file 3.**

## Data Availability

All data generated and analysed during this study are included in this published article and its supplementary information files.

## Abbreviations

MCAR: Missing completely at random
MAR: Missing at random
MNAR: Missing not at random
IQR: Interquartile range
