# Supplementary material for: The reporting and handling of missing data in longitudinal studies of older adults is suboptimal: a methodological survey of geriatric journals
Source: BMC Med Res Methodol. 2022 Apr 26;22:122. doi: 10.1186/s12874-022-01605-w (PMC9040343; doi:10.1186/s12874-022-01605-w)
Supplement: Supplementary file 1 — Additional file 1. [file 12874_2022_1605_MOESM1_ESM.docx]

Supplementary file 1: Search strategy

Medline Search

| 1 | Aged/ | 3,337,283 |
| --- | --- | --- |
| 2 | "Aged, 80 and over"/ | 1,003,928 |
| 3 | Aging/ | 243,738 |
| 4 | ag?ing.ti,ab. | 248,528 |
| 5 | advanced years.ti,ab. | 73 |
| 6 | (old* adj3 (age or m?n or male** or wom?n or female* or people or adult* or population or person*)).ti,ab. | 827,155 |
| 7 | pensioner.ti,ab. | 184 |
| 8 | late?life.ti,ab. | 9 |
| 9 | elder*.ti,ab. | 282,267 |
| 10 | retire*.ti,ab. | 23,794 |
| 11 | senior*.ti,ab. | 46,613 |
| 12 | exp Geriatrics/ | 31,031 |
| 13 | geriatric*.ti,ab. | 53,523 |
| 14 | post?menopausal women.ti,ab. | 38,432 |
| 15 | 1 or 2 or 3 or 5 or 6 or 7 or 8 or 11 or 12 or 13 or 14 | 4,140,323 |
| 16 | (exp infant/ or exp child/ or adolescent/) not exp adult/ | 2,032,508 |
| 17 | 15 not 16 | 4,091,570 |
| 18 | exp longitudinal studies/ | 156,484 |
| 19 | exp prospective studies/ | 620,071 |
| 20 | exp retrospective studies/ | 1,007,856 |
| 21 | longitudinal stud*.ti,ab. | 87,125 |
| 22 | retrospective stud*.ti,ab. | 191,947 |
| 23 | prospective stud*.ti,ab. | 192,672 |
| 24 | follow?up.ti,ab. | 21,096 |
| 25 | exp cohort studies/ | 2,317,275 |
| 26 | cohort stud*.ti,ab. | 266,653 |
| 27 | Observational Study/ | 123,735 |
| 28 | observational stud*.ti,ab. | 136,895 |
| 29 | cohort analysis.ti,ab. | 9,116 |
| 30 | Epidemiologic Studies/ | 9,043 |
| 31 | epidemiological stud*.ti,ab. | 63,255 |
| 32 | 18 or 19 or 20 or 21 or 22 or 23 or 24 or 25 or 26 or 27 or 28 or 29 or 30 or 31 | 2,691,996 |
| 33 | 17 and 32 | 1,087,861 |
| 34 | limit 33 to yr="2015 - 2019" | 305,061 |
| 35 | limit 34 to english language | 297,698 |
| 36 | (Age & Ageing or BMC Geriatrics or Aging & Disease or Journal of the American Geriatrics Society or Journals of Gerontology Series A-Biological Sciences & Medical Sciences or Geroscience or Journal of the American Medical Directors Association or Journal of Aging & Health or Clinical Interventions in Aging or Aging-Clinical & Experimental Research).jn. | 48,297 |
| 37 | 35 and 36 | 3,580 |

Embase Database

| 1 | aged/ | 3,323,061 |
| --- | --- | --- |
| 2 | elder*.ti,ab. | 398,796 |
| 3 | elderly care/ | 41,582 |
| 4 | ag?ing.ti,ab. | 313,317 |
| 5 | aging/ | 287,177 |
| 6 | geriatrics/ | 32,097 |
| 7 | geriatric*.ti,ab. | 84,336 |
| 8 | advanced years.ti,ab. | 100 |
| 9 | (old* adj3 (age or m?n or male** or wom?n or female* or people or adult* or population or person*)).ti,ab. | 1,188,542 |
| 10 | senior*.ti,ab. | 65,372 |
| 11 | pensioner*.ti,ab. | 1,331 |
| 12 | late?life.ti,ab. | 110 |
| 13 | post?menopausal women.ti,ab. | 54,159 |
| 14 | 1 or 2 or 3 or 4 or 5 or 6 or 7 or 8 or 9 or 10 or 11 or 12 or 13 | 4,548,987 |
| 15 | (exp infant/ or exp child/ or adolescent/) not exp adult/ | 2,323,648 |
| 16 | 14 not 15 | 4,470,674 |
| 17 | longitudinal study/ | 169,682 |
| 18 | prospective study/ | 754,272 |
| 19 | retrospective study/ | 1,219,298 |
| 20 | longitudinal stud*.ti,ab. | 112,286 |
| 21 | retrospective stud*.ti,ab. | 304,280 |
| 22 | prospective stud*.ti,ab. | 291,565 |
| 23 | follow up/ | 1,815,591 |
| 24 | follow?up stud*.ti,ab. | 1,537 |
| 25 | cohort analysis/ | 821,031 |
| 26 | cohort stud*.ti,ab. | 385,666 |
| 27 | 17 or 18 or 19 or 20 or 21 or 22 or 23 or 24 or 25 or 26 | 3,917,899 |
| 28 | 16 and 27 | 1,203,084 |
| 29 | limit 28 to yr="2015 - 2019" | 418,813 |
| 30 | limit 29 to english language | 412,038 |
| 31 | (Age & Ageing or BMC Geriatrics or Aging & Disease or Journal of the American Geriatrics Society or Journals of Gerontology Series A-Biological Sciences & Medical Sciences or Geroscience or Journal of the American Medical Directors Association or Journal of Aging & Health or Clinical Interventions in Aging or Aging-Clinical & Experimental Research).jn. | 39,526 |
| 32 | 30 and 31 | 3,452 |
