# Supplementary material for: The reporting and handling of missing data in longitudinal studies of older adults is suboptimal: a methodological survey of geriatric journals
Source: BMC Med Res Methodol. 2022 Apr 26;22:122. doi: 10.1186/s12874-022-01605-w (PMC9040343; doi:10.1186/s12874-022-01605-w)
Supplement: Supplementary file 3 — Additional file 3. [file 12874_2022_1605_MOESM3_ESM.docx]

**Supplementary file 3: Missing Data Reporting**

Were there any missing data?

No Yes

Report data were complete

Report amount of missing data per variable, per analysis, and for entire sample

Indicate reasons for missing data

Report results of comparison between participants with and without missing data

State the method used to handle missing data

Describe the assumptions made for missing data analysis (e.g., MCAR, MAR, MNAR)

If multiple imputation was used

Report the results of primary analysis and sensitivity analysis of missing data

Describe how non-normal or categorical variables were handled

Report the number of variables included in the model

Indicate the number of imputations

^1^Vandenbroucke JP, Von Elm E, Altman DG, Gøtzsche PC, Mulrow CD, Pocock SJ, et al. Strengthening the Reporting of Observational Studies in Epidemiology (STROBE): Explanation and elaboration. PLoS Med. 2007;4(10):1628–54.

^2^Sterne JAC, White IR, Carlin JB, Spratt M, Royston P, Kenward MG, et al. Multiple imputation for missing data in epidemiological and clinical research: Potential and pitfalls. BMJ. 2009;339(7713):157–60.

Report any evaluation of the imputation process

**Flow Chart for Reporting of Missing Data^1,2^**

**Example 1: Study with no missing data** (1)

“The complete data represent 188 full observations of 60 variables (no missing data)”.

**Example 2: Study with missing data** (2)

**Main article**

Missing data per variable were reported in the table describing the sample characteristics.

“Multiple imputation using a full conditional specification model (chained equation) in the analysis was used to account for missing data, which was mainly due to the interview being conducted through a proxy or the 1905 cohort member being physically or mentally unable to perform the tests.22 Online Appendix S1 describes the multiple imputation in detail”.

**Supplementary file**

“Multiple imputation was performed using a full conditional specification model (chained equation) with ten iterations of the burn-in period. It was examined whether the ten iterations were adequate for the chain to converge to a stationary distribution. To minimize the Monte Carlo error, 50 imputation sets were generated. In this procedure a series of regression models are run whereby each variable with missing data is modeled conditional upon the other variables in the data. This means that each variable can be modeled according to its own distribution. In the imputation model of each variable all the other measures were used to impute the missing value including the remaining life span which was log transformed. The imputation model was performed separately for each gender, i.e. not assuming similar association between the measures and mortality for both genders. Multiple imputation has the advantage that it only requires the data to be missing at random (MAR) to give an unbiased result whereas using complete case analysis assumes missing completely at random or at least that the missing data are caused only by the measure (exposure) itself.15, 16 However, since mortality (outcome) adjusting for the measure still predicts the missing data, complete case analysis would give a biased result. Besides the measures and mortality, additional (auxiliary) variables from the health survey were also used in the imputation model to address the assumption that the data are missing at random which is the assumption of multiple imputation. Auxiliary variables are variables that are predictive of the missingness of the variable in question. Since the missing data was due mainly to the interview being through a proxy, variables which were both found in the non-proxy and proxy interviews were most important to find to address the MAR. The auxiliary variables were mainly found by automated stepwise procedure. All 6 variables were given the same attention in the imputation model, however since the variables about socioeconomic factors, disease, medication, and activity of daily living had very few missing data the imputation will have little impact on the analysis of these variables. Hence, we will in the following turn our attention to the imputation of the variables about physical performance, cognition and general and mental health perception. Missing values of the chair stand were imputed using an ordered logistic regression and some of the auxiliary variables which were used were information about if they could wash their lower part of their body, if they have had a fracture of their femur or if they used a wheel chair or walker. Missing values of grip strength were imputed using a linear regression with a bound between 1 and 60 to be sure to create valid imputations. Some of the auxiliary variables used in the imputation of the grip strength were information about if they lived in a nursing home or if they had visit from a home care service. Missing information of being able to walk was imputed by a logistic regression and auxiliary variables such as information if they used a wheelchair, a walker or a cane. Missing values of the walking speed were imputed by a linear regression with a bound between 2 and 30 seconds. It was only imputed if being able to walk was imputed as yes and information about if they had fallen within the last 6 month or if they used a walker was some of the variables used as auxiliary variables. Before the imputation, walking speed was transformed to be more normally distributed and after the imputation model it was transformed back by the inverse function. Missing values of the MMSE were imputed using a linear regression with a bound between 0 and 30 and MMSE scores were transformed to be more normally distributed. Auxiliary variables such as information about if they had a hobby, if they had visit from a home care service, or if they were senile was used in the imputation. Information about senility of the 1905 cohort member was either 7 informed from the proxy or from the interviewer who recorded if the interview was to some degree difficult to perform because the respondent seemed to be senile. Missing values for the five tests which comprises the cognitive composite score was imputed separately and from this the cognitive composite score was formed. Appropriate regression model was used for the five tests and auxiliary variables used for these tests were almost the same as those used in the imputation model for MMSE.”

1. Level C, Tellier E, Dezou P, Chaoui K, Kherchache A, Sejourné P, et al. Outcome of older persons admitted to intensive care unit, mortality, prognosis factors, dependency scores and ability trajectory within 1 year: a prospective cohort study. Aging Clin Exp Res [Internet]. 2018;30(9):1041–51. Available from: http://dx.doi.org/10.1007/s40520-017-0871-z

2. Thinggaard M, McGue M, Jeune B, Osler M, Vaupel JW, Christensen K. Survival Prognosis in Very Old Adults. J Am Geriatr Soc. 2016;64(1):81–8.
